# Design Choices for Next-Generation Neurotechnology Can Impact Motion Artifact in Electrophysiological and Fast-Scan Cyclic Voltammetry Measurements

**DOI:** 10.3390/mi9100494

**Published:** 2018-09-27

**Authors:** Evan N. Nicolai, Nicholas J. Michelson, Megan L. Settell, Seth A. Hara, James K. Trevathan, Anders J. Asp, Kaylene C. Stocking, J. Luis Lujan, Takashi D.Y. Kozai, Kip A. Ludwig

**Affiliations:** 1 Mayo Clinic Graduate School of Biomedical Sciences, Rochester, MN 55905, USA; nicolai.evan@mayo.edu (E.N.N.); settell.megan@mayo.edu (M.L.S.); trevathan.james@mayo.edu (J.K.T.); asp.anders@mayo.edu (A.J.A.); 2 Department of Bioengineering, University of Pittsburgh, Pittsburgh, PA 15213, USA; njm89@pitt.edu (N.J.M.); kcs58@pitt.edu (K.C.S.); 3 Department of Psychiatry, University of British Columbia, Vancouver, BC V6T 1Z3, Canada; 4 Division of Engineering, Mayo Clinic, Rochester, MN 55905, USA; hara.seth@mayo.edu; 5 Department of Neurologic Surgery, Mayo Clinic, Rochester, MN 55905, USA; lujan.luis@mayo.edu; 6 Department of Physiology and Biomedical Engineering, Mayo Clinic, Rochester, MN 55905, USA; 7 Center for the Neural Basis of Cognition, University of Pittsburgh and Carnegie Mellon University, Pittsburgh, PA 15213, USA; 8 McGowan Institute for Regenerative Medicine, University of Pittsburgh, Pittsburgh, PA 15213, USA; 9 NeuroTech Center of the University of Pittsburgh Brain Institute, Pittsburgh, PA 15213, USA; 10 Center for Neuroscience, University of Pittsburgh, Pittsburgh, PA 15213, USA; 11 Department of Bioengineering, University of Wisconsin, Madison, WI 53706, USA; 12 Department of Neurological Surgery, University of Wisconsin, Madison, WI 53706, USA

**Keywords:** electrode, artifact, electrophysiology, electrochemistry, fast-scan cyclic voltammetry (FSCV), neurotechnology, neural interface, neuromodulation, neuroprosthetics, brain-machine interfaces

## Abstract

Implantable devices to measure neurochemical or electrical activity from the brain are mainstays of neuroscience research and have become increasingly utilized as enabling components of clinical therapies. In order to increase the number of recording channels on these devices while minimizing the immune response, flexible electrodes under 10 µm in diameter have been proposed as ideal next-generation neural interfaces. However, the representation of motion artifact during neurochemical or electrophysiological recordings using ultra-small, flexible electrodes remains unexplored. In this short communication, we characterize motion artifact generated by the movement of 7 µm diameter carbon fiber electrodes during electrophysiological recordings and fast-scan cyclic voltammetry (FSCV) measurements of electroactive neurochemicals. Through in vitro and in vivo experiments, we demonstrate that artifact induced by motion can be problematic to distinguish from the characteristic signals associated with recorded action potentials or neurochemical measurements. These results underscore that new electrode materials and recording paradigms can alter the representation of common sources of artifact in vivo and therefore must be carefully characterized.

## 1. Introduction

Implantable neural interface devices can be used to examine chemical or electrical activity within the brain, making them critical tools for conducting neuroscience research [1,2,3,4,5,6,7]. The information recorded from these instruments can also enable state-of-the-art therapeutic or rehabilitative strategies [8]. For example, extracellular neuronal signal information recorded from implanted microelectrode arrays (i.e., single units, multiunits, or local field potentials) may be used to decode intended movements and control assistive devices [1,9,10,11,12]. Similarly, measurements of phasic changes in the extracellular concentration of dopamine taken by small electrodes placed within the brain have been proposed as a feedback signal to titrate levels of deep brain stimulation (DBS) to alleviate tremors associated with Parkinson’s Disease [13,14,15]. 

The utility of a neural interface device depends on how well one can differentiate the measured physiological signal of interest from other sources of physiological signals (electromyogram (EMG), electrooculogram (EOG)), noise (shot noise, flicker noise, etc.), and artifact signals [16]. Artifact signals that resemble physiological signals—caused by motion [17], stimulation [18], or changes in ambient radiofrequency (RF) noise [19]—are a known problem for in vivo electrophysiological recordings. Motion artifacts are particularly troublesome because they can be highly variable in amplitude across time, making them difficult to remove with standard filtering techniques and therefore potentially difficult to distinguish from the measurands of interest [17,20].

There are three primary mechanisms by which a motion artifact can be generated. First, motion experienced during free behavior is often transferred to connection points, such as where external instrumentation is plugged into a head-stage on the animal. Atoms interact at the boundary point where two objects touch each other, generating a charge at both material interfaces. When these objects move relative to each other, an electrostatic voltage is generated known as the triboelectric effect [21]. Second, motion of the wire induces an artifact current if the wire is subject to intrinsic or extrinsic magnetic fields [22]. Lastly, artifact can be introduced by motion of the electrode with respect to the neural tissue at the electrode/electrolyte interface [20]. When an electrode is placed in an electrolytic solution, a multitude of events takes place. Solvent molecules adsorb to the surface, resulting in the formation of an electric double-layer; non-specifically adsorbed ions also form a diffuse layer, which is dependent on the ionic concentration of the solution. A potential difference between the electrode and electrolyte is also generated by oxidation/reduction electrochemical reactions occurring at the surface of the electrode [17,23]. Electrochemical reactions continue until the potential difference between the electrode and the electrolyte drives an equal balance between oxidation and reduction reactions to generate no net current at the interface [23,24]. When an electrode is moved with respect to the electrolyte, equilibrium at the electrode/electrolyte interface is disturbed and must be reestablished, creating an artifact current [25].

Recently, there has been increased interest in developing 7 µm diameter carbon fiber electrodes for chronic electrophysiological recordings and measurements of electroactive neurochemicals in vivo using fast-scan cyclic voltammetry (FSCV) [26,27,28,29,30,31,32,33,34,35,36,37,38,39,40,41,42,43,44]. The small diameter limits the amount of tissue displaced during implantation, mitigating local neurodegeneration and damage to synaptic sources of neurotransmitters of interest [30,44,45]. Moreover, carbon fibers can be manufactured to be flexible post-implantation, improving the mechanical match with surrounding brain tissue and thereby decreasing chronic inflammation [28,29,30,32,33,40]. Although initial results utilizing ultra-small, flexible carbon fiber electrodes are promising, the impact of these design changes on the representation of motion artifact during recording remains largely unexplored. 

Here we demonstrate on the benchtop and in vivo that motion of carbon fibers while recording can generate artifact signals that are nearly indistinguishable from neural electrophysiological or neurochemical signals. In addition, classical signal processing techniques can exacerbate the similarity of these artifact signals to physiological signals. These findings highlight that one must carefully consider how neural interface design changes impact unwanted artifact signals in behaving neural recordings, which ultimately may lead to novel neural interface designs that minimize the impact of these artifacts [20,28]. 

## 2. Materials and Methods 

### 2.1. Electrophysiological Recordings

All experimental protocols were approved by the University of Pittsburgh Division of Laboratory Animal Resources and Institutional Animal Care and Use Committee, in accordance with the standards for humane animal care as set by the Animal Welfare Act and the National Institutes of Health Guide for the Care and Use of Laboratory Animals.

Benchtop data was collected from a single channel microwire, placed in a three-electrode electrochemical cell (1× phosphate buffered saline (PBS), AgCl reference, Pt Counter electrode). Recordings were conducted with the microwire within a fully electrically isolated Faraday cage. To simulate motion, the wire was moved three times using a non-conductive zip tie over the course of the recording duration (approximately 8 s). To compare benchtop data with in vivo results, C57BL/6 mice (22–28 g) were implanted with a planar, silicon microelectrode array (A-1×16-3 mm-100-703-CM16LP, Neuronexus Technologies. Ann Arbor, MI, USA) in left monocular visual cortex [6,20,32,46,47]. Additionally, artifacts were compared between flexible carbon fiber arrays and rigid silicon arrays, with simultaneous recordings from an adult male Long Evans rat implanted with a 16 channel carbon fiber array in right primary motor cortex, and a single shank silicon electrode array in left primary motor cortex [29,40]. In each recording session, animals were awake, non-behaving, and non-restrained [20,29,40]. Recordings were conducted from within a fully electrically isolated Faraday cage. Data was sampled at 24,414 Hz, with a pre-amplifier high pass filter at 2.2 Hz and an anti-aliasing filter at 7.5 kHz. The raw data was then filtered using a 2nd order Butterworth filter (300–5000 Hz) to produce spike streams. A threshold set at 3.5 standard deviations below the mean of each channel’s spike stream data was established for the detection of single or multiunit activity. Putative electrophysiological artifacts were identified using a custom MATLAB script (MATLAB R2016b, MathWorks, Inc., Natick, MA, USA), as previously described [20]. Briefly, artifacts were defined as incidents in which threshold crossing events occurred simultaneously (±0.05 ms) across at least three channels. As the voltage of the extracellular action potential decays substantially with distance [28,48], threshold crossing events which occurred simultaneously across three channels are unlikely to be caused by a single neuron, given the 100 µm spacing between electrode sites [49]. As expected, in corresponding anesthetized studies, threshold crossings were not detected on multiple channels within 0.05 ms of each other [5,6,7,20,32,46,47,50]. Additionally, although certain neurophysiologic events such as spindle activity [51] may be observed across such distances, lower frequency activity (<300 Hz) was filtered before analyzing spike trains.

### 2.2. Electrochemical Recordings

Carbon fiber FSCV microelectrodes were built in-house, based on the electrodes described by Clark et al. [44]. Briefly, a single 7 µm diameter carbon fiber was placed within a silica tube of 100 µm diameter and one end was sealed with polyimide. The other end was connected to an extension wire of 300 µm diameter nitinol using an equal parts 99% pure silver powder and polyimide mixture. The exposed carbon fiber was trimmed to a final length of 100 µm. Reference electrodes (Ag-AgCl) were constructed using a silver wire immersed in 0.9% NaCl solution and applying a current using a 9 V battery to form a chlorinated electrode. 

To test the carbon fiber electrodes in vitro, multiple experiment vessels—50 mL Falcon tubes—were filled with 0.6% agarose in tris-buffered saline (140 mM NaCl, 1.25 mM NaH_2_PO_4_, 3.25 mM KCl, 1.2 mM CaCl_2_, 15 mM Trizma Base, 1.2 mM MgCl_2_, 2 mM Na_2_SO_4_) to create an agarose gel solution that mimics the viscoelastic properties of the brain [52,53]. The concentrations of dopamine in each gel were varied to characterize motion artifact during FSCV recordings as a function of dopamine concentration in solution. All chemicals were purchased from Sigma-Aldrich (St. Louis, MO, USA).

**Figure 1:**
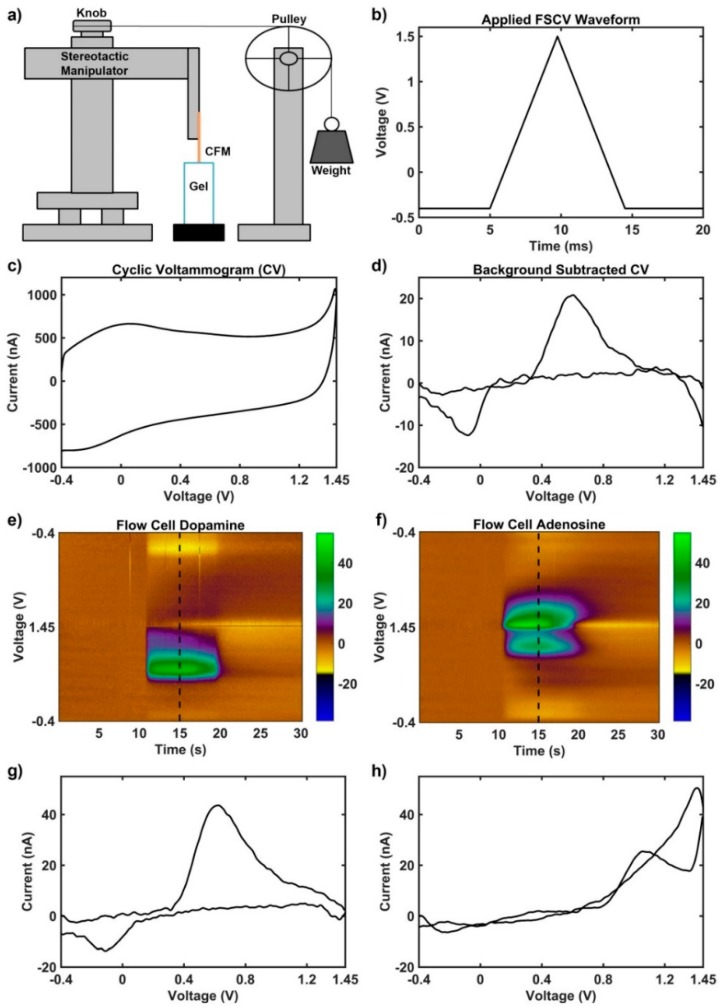
Experimental setup and fast scan cyclic voltammetry methodology. (**a**) Schematic of the mechanism used to produce motion of the carbon fiber microelectrode through agar solution without generating electromagnetic interference. (**b**) Example triangular voltage waveform applied to carbon fiber microelectrode vs. the Ag-AgCl reference electrode. (**c**) Example current obtained at all voltage points during application of triangle waveform (cyclic voltammogram) in (**b**). The current generated includes both capacitive and faradaic components. (**d**) Example cyclic voltammogram obtained via background subtraction during a transient dopamine concentration change, which emphasizes faradaic changes such as the oxidation near 0.6 V and the reduction near −0.2 V. (**e**–**h**) Known signals for dopamine and adenosine obtained via measurement in a benchtop flow cell (different than the setup in Figure 1A) using the methodology described by Shon et al. [54]. Briefly, a flow cell passes buffer solution by the electrode continuously with transient boluses of electrochemical in order to produce a phasic signal. High concentrations (1 μM dopamine and 5 μM adenosine) were passed by the carbon fiber microelectrode in pure buffer to produce large, idealistic—low noise—signals to emphasize the characteristic faradaic components (**e**) Dopamine signal with characteristic oxidation at 0.6 V and reduction at −0.2 V. (**g**) Characteristic cyclic voltammogram of dopamine signal from (**e**) (collected at dotted vertical line). (**f**) Adenosine signal with characteristic primary oxidation at 1.4 V and secondary oxidation at 1.0 V. (**h**) Characteristic cyclic voltammogram of adenosine signal from (**f**) (collected at dotted vertical line).

A novel experimental set-up was devised to generate controlled movement of the carbon fiber in the agarose solution without the use of a power source which might also induce artifact. A stereotactic manipulator knob controlling movement in the vertical plane was connected to a weight hanging from a pulley (Figure 1a). This was done such that when the weight was released, the stereotactic knob would turn and move the electrode down a set distance. A non-conductive zip tie was used to turn the stereotactic knob, which lifted the electrode up through the solution. Releasing the weight would then lower the electrode a set distance via free fall acceleration by gravity. Carbon fiber microelectrodes were placed in a holder secured to this stereotactic manipulator, then initially lowered into the gel solution such that the electrode tip was centered and at approximately the 25 mL mark on the Falcon tube (half the depth).

All experiments were performed in a fully electrically isolated Faraday cage. Data was collected using the Universal Electrochemical Instrument (UEI, University of North Carolina, Chapel Hill, NC, USA) starting with a gel solution that had no neurochemicals, and proceeding through multiple gel solutions containing various concentrations of neurochemical in a randomized order (2, 5, 10 µM dopamine, *n* = 6 electrodes and *n* = 3 of these electrodes included 1 µM dopamine; 10, 20, 50 µM adenosine, *n* = 1 electrode). The applied voltage waveform was held at −0.4 V with triangular excursions to 1.3 V and back at a 400 V/s scan rate every 100 milliseconds for measurements of dopamine; a similar waveform was used for measurements of adenosine, except the peak voltage was 1.5 V instead of 1.3 V. Within each solution, the carbon fiber microelectrode was subjected to a slow upward motion lasting approximately 3 s, a 30 s motionless period, and then a fast downward motion (~0.250 s) resulting from the free fall of a hanging weight (147 g). Duration of the motion was measured using a slow motion camera, and the duration (0.25 s) and distance traveled (1 mm) were used to calculate the acceleration (0.032 m/s^2^). A minimum of six measurements were collected per concentration; three measurements were taken during 100 µm movements and three during 1 mm movements to mimic the distance traveled by the brain during behavior in rodents and non-human primates, respectively [46].

Data was analyzed using HDCV Analysis (University of North Carolina, Chapel Hill, NC, USA). Filtering and automatic averaging were turned off. The average of five cyclic voltammograms occurring one second before motion occurred was used for background subtraction. A background subtracted cyclic voltammogram collected 200 milliseconds after the start of the motion was used to determine location of peak oxidation potential. An average of the peak current at the oxidation potential and the two data points before and after the peak following application of motion was recorded as the magnitude of the artifact response.

## 3. Results

Movement can generate robust electrical artifact signal through (1) the triboelectric effect, (2) electromagnetic flux magnified by active electronics, and (3) disruption of the electrochemical interface. In the following section, motion artifact is first characterized during electrophysiological recordings, where active electronics are used to minimize current flow. Then, the impact of motion artifact is characterized during FSCV neurochemical recordings for comparison. During FSCV a triangle waveform is applied to oxidize/reduce neurochemicals adsorbed to the carbon fiber electrode surface, adding additional complexity to the recording system. 

### 3.1. Motion Artifact During Electrophysiological Recordings

**Figure 2:**
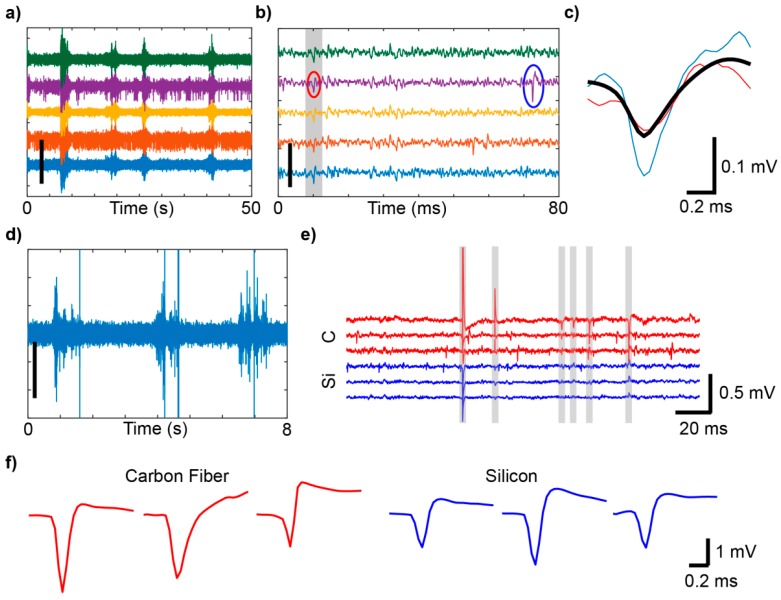
Animal movement creates artifacts that contaminate electrophysiological recordings. (**a**) Sample recordings from a silicon electrode array implanted in an awake, freely moving mouse. (**b**) Same as (**a**), over 80ms. Gray shading highlights artifacts. A true action potential is circled in blue, while an artifact recorded on the same channel is circled in red. (**c**) Example spike and artifact waveforms from (**b**). In this case, principal component analysis (PCA) and *k*-means clustering failed to separate the highlighted artifact (red) from the action potential (blue). The cluster average (black), as isolated with PCA and *k*-means clustering, is shown for comparison. (**d**) Brief recordings from an un-implanted microwire, where the electrode was moved three times with a non-conductive zip tie. (**e**) Sample recording data taken simultaneously from a carbon fiber array (red) and a silicon array (blue) implanted in right and left primary motor cortex in a rat, respectively. Gray shading highlights artifacts that were flagged using the detection method described previously. (**f**) Examples of artifacts from the carbon fiber array (red) and silicon array (blue). Scalebars: (**a**,**b**) 400 µV, (**c**,**d**) 100 µV. (**a**–**e**): © IOP Publishing. Adapted with permission from [20]. All rights reserved.

Electrophysiological recordings are susceptible to contamination by artifacts, which can appear as high amplitude voltage deflections that occur simultaneously across multiple channels (Figure 2a). These non-neural signals demonstrate high variability in shape and amplitude, and in some cases, may share similar temporal characteristics to single unit or multiunit signals (Figure 2b). These similarities may result in the misclassification of those artifacts as neural signals. In our awake free-moving mouse recordings, principal component analysis and *k*-means clustering failed to separate all artifacts from action potentials, resulting in the grouping of some artifact signals with single units (Figure 2c red and blue snippets, respectively). For comparison with in vivo data recordings, artifacts were also generated on the benchtop without an animal, using an un-implanted single channel microwire connected through the same headstage preamplifier and amplifier in a three electrode electrochemical cell in 1× PBS placed in a fully electrically isolated Faraday cage. The electrode was moved with a non-conductive zip tie held outside of the Faraday cage. This ensured that electrical artifacts or line noise originating from outside of the cage did not influence the signal obtained from within the cage. Movement of the electrode three times generated high amplitude artifacts (Figure 2d) [20]. In awake free-moving rats, recordings were conducted simultaneously from a 16 channel carbon fiber array and a single shank silicon electrode array implanted into the right and left primary motor cortex, respectively [29]. Here, artifacts were more prominent on the carbon fiber array (Figure 2e,f, red traces) than the silicon array (Figure 2e,f, blue traces), suggesting that differences in the design of the carbon fiber array contributed to the representation of motion artifact.

### 3.2. Motion Artifact During FSCV Recordings

**Figure 3:**
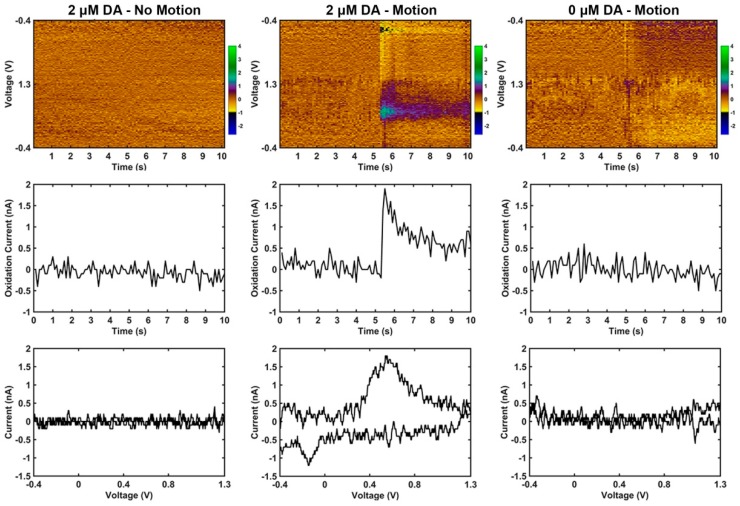
Motion causes dopamine-like artifact signal in vitro only in the presence of dopamine (DA); data is from one representative carbon fiber microelectrode. **Top row:** Pseudo color plots where x, y, and z are time (s), voltage (V), and current (nA) respectively. **Middle row:** Current vs. time plots at the known 0.6 V oxidation potential of dopamine. **Bottom row:** Current vs. voltage plots (cyclic voltammograms) collected 200 μs following initiation of motion. For the no-motion condition, the cyclic voltammogram was collected at the 5 s mark. **Left column:** Example carbon fiber microelectrode FSCV recordings in 0.6% agarose with 2 μM dopamine in solution when no motion is applied. There are no artifact signals apparent in the color plot. **Middle column:** Example of the same electrode in the same solution as the left column data but with a 1 mm motion downward at the 5 s mark that lasts approximately 0.25 s. Note the presence of a transient signal following motion that has the characteristic oxidation and reduction potential changes of dopamine. **Right column:** Example of the same electrode as the other columns in a gel solution with no dopamine and a 1 mm motion downward at the 5 s mark that lasts approximately 0.25 s. There is a transient, broad band disturbance in the color plot at the time of motion, but with no apparent changes at specific voltages.

**Figure 4:**
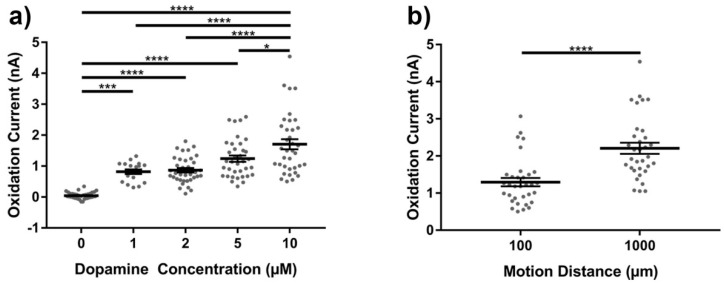
Characterization of fast-scan cyclic voltammetry (FSCV) motion artifacts in vitro in gel solutions of dopamine. (**a**) Effect of gel solution dopamine concentration on motion evoked artifact current at the dopamine oxidation potential 0.6 V (all data points shown as grey dots, mean ± standard error of the mean shown as horizontal black lines and error bars); 0 μM–0.044 ± 0.018 nA (mean ± standard error, *n* = 6 electrodes, 36 measurements), 1 μM–0.816 ± 0.075 nA (*n* = 3 electrodes, 18 measurements), 2 μM–0.865 ± 0.071 nA (*n* = 6 electrodes, 36 measurements), 5 μM–1.241 ± 0.103 nA (*n* = 6 electrodes, 36 measurements), 10 μM–1.706 ± 0.164 nA (*n* = 6 electrodes, 36 measurements); * indicates *p*-value < 0.05, *** *p*-value < 0.005, **** *p*-value < 0.0001, 1 μM vs. 2 μM was non-significant (*p*-value > 0.999), 2 μM vs. 5 μM was non-significant (*p*-value = 0.082), 1 μM vs. 5 μM was non-significant (*p*-value = 0.145), one way ANOVA with post-hoc Bonferroni’s multiple comparisons test). (**b**) Effect of motion distance on motion evoked artifact current at the dopamine oxidation potential 0.6 V in gel solutions containing 10 μM dopamine (all data points shown as grey dots, mean ± standard error of the mean shown as horizontal black lines and error bars); 100 µm–1.294 ± 0.112 nA (mean ± standard error, *n* = 6 electrodes, 33 measurements), 1000 µm–2.206 ± 0.148 nA (*n* = 6 electrodes, 33 measurements); **** indicates *p*-value < 0.0001, *t*-test with Welch’s correction).

The representation of motion artifact during FSCV recordings has not previously been characterized. As described in the methods, it was important to ensure that electromagnetic radiation from motors and pumps did not influence the motion artifact; therefore, a pulley and weight system was used to generate the motion. Phasic changes in dopamine concentration in vivo generate corresponding changes in currents measured at 0.6 V and −0.2 V during FSCV recordings, due to the oxidation and reduction of dopamine respectively. During motion, transient signals were reliably produced with current changes observed at the characteristic oxidation and reduction potentials for dopamine (DA), but only when dopamine was in solution (Figure 3, center column). Without DA in solution, changes in current due to motion were often not detectable or appeared as low amplitude, broad changes that bear no resemblance to neurochemicals of interest (Figure 3, right column). FSCV data was also collected in the absence of motion, and does not contain apparent artifacts (Figure 3, left column). The amplitude of the artifact signal was dependent on both the concentration of dopamine in the experiment vessel (Figure 4a) and the distance traveled by the microelectrode (Figure 4b). 

**Figure 5:**
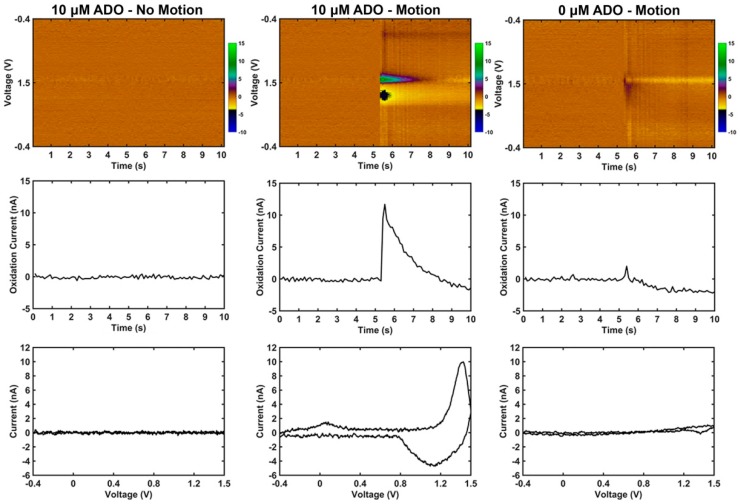
Motion causes adenosine-like artifact signal in vitro only in the presence of adenosine (ADO) that is different than motion induced artifact produced in the presence of dopamine; data is from one representative carbon fiber microelectrode. **Top row:** Pseudo color plots where x, y, and z are time (s), voltage (V), and current (nA) respectively. **Middle row:** Current vs. time plots at the known 1.4 V primary oxidation potential of adenosine. **Bottom row:** Current vs. voltage plots (cyclic voltammograms) collected 200 μs following initiation of motion. For the no-motion condition, the cyclic voltammogram was collected at the 5 s mark. **Left column:** Example carbon fiber microelectrode FSCV recordings in 0.6% agarose with 10 μM adenosine in solution when no motion is applied. There are no artifact signals apparent in the color plot. **Middle column:** Example of the same electrode in the same gel solution as the left column but with a 1 mm motion downward at the 5 s mark that lasts approximately 0.25 s. Note the increase in current at 1.4 V resembling that of the known primary oxidation for ADO signal (Figure 1f), and a decrease in current near the known secondary oxidation for ADO. **Right column:** Example of the same electrode as the other columns in a gel solution containing no adenosine and a 1 mm motion downward at the 5 s mark that lasts approximately 0.25 s. There is a transient, broad band change at the time of motion, but the signal possesses no apparent changes at specific voltages.

To determine whether the artifact signal was specific to dopamine, similar motion in the presence of adenosine was also evaluated (Figure 5). In contrast to dopamine, adenosine adsorbed to the surface of the electrode generates a primary oxidation peak at 1.4 V and a secondary oxidation peak at 1.0 V during FSCV (Figure 1f) [55]. Due to the kinetics of the reaction, the secondary oxidation peak is often delayed from the onset of the primary oxidation peak by 0.5 s during a phasic change in adenosine [55]. Notably, the motion artifact in dopamine gel solution (Figure 3, center column) strongly resembles phasic change in dopamine concentration (Figure 1e). In contrast, the motion artifact in adenosine gel solution (Figure 5, center column) has an increase in current at 1.4 V matching the primary oxidation peak observed during phasic change in adenosine concentration, but an apparent decrease in current near 1.0 V. This is the opposite direction of current changes expected at the secondary oxidation peak during phasic changes of adenosine (Figure 1f).

## 4. Discussion

The data presented in this short communication show that motion can generate electrophysiological and electrochemical artifacts resembling signals traditionally associated with physiological changes of interest. Movement can generate robust artifact signal through numerous potential mechanisms. While the current work does not isolate the individual contribution of each potential mechanism, this work highlights the necessity and importance of device and experimental design to accurately interpret the detected signals. The present work characterizes the impact of motion artifact signals on electrophysiological and neurochemical recordings, as well as emphasizes the existence of an unexpected trade-off—potentially enhanced motion artifact—inherent to developing flexible devices for electrophysiological and neurochemical sensing.

### 4.1. Motion Artifact During Electrophysiological Recordings Using Flexible Carbon Fibers

The electrode system used for recordings in this study may have impacted the manifestation of motion artifact in several ways. First, in contrast to comparatively more rigid silicon arrays, small diameter carbon fibers are manufactured with geometries that give them greater compliancy [30]. More compliant electrodes are more likely to bend when subjected to stress [26,31,35,36,37,38,39]. The bending of the wire can in turn generate electrostatic and electromagnetic artifact current that can be exacerbated by active electronics [22,56]. 

Electrophysiological recordings require the use of preamplifier headstage/amplifier stages, which may also be a source of motion artifact signals [16]. In an ideal recording system, small electrical potentials are amplified by the preamplifier headstage and amplifier prior to digitization [56]. The preamplifier is placed as close to the electrode as possible to minimize the length of wire prior to amplification, which minimizes noise introduced by coupling between extrinsic noise sources and the length of wire. Often a small bundle of wire within the headstage still connects the preamplifier output to the amplifier input for subsequent analog to digital conversion. Because the wire is part of the active electronics of the headstage, electrical current is being maintained through the headstage, electrode, the reference, and ground. Each point of connection represents a potential source of triboelectric artifact. These results have implications for integrated multiplexers (MUX) on high-channel recording arrays and on-array powered electronics. Switching across MUX channels requires changes in current to switch channels, which capacitively couples with and leads to charge injection in the analog signal path [57]. Switching also disturbs the equilibrium potential of the electrode/electrolyte interface as a previously floating electrode is connected to the measurement circuit [57]. This issue has the potential to limit the frequency at which channels can be sampled when using multiplexers for electrophysiological and neurochemical signal detection.

**Figure 6:**
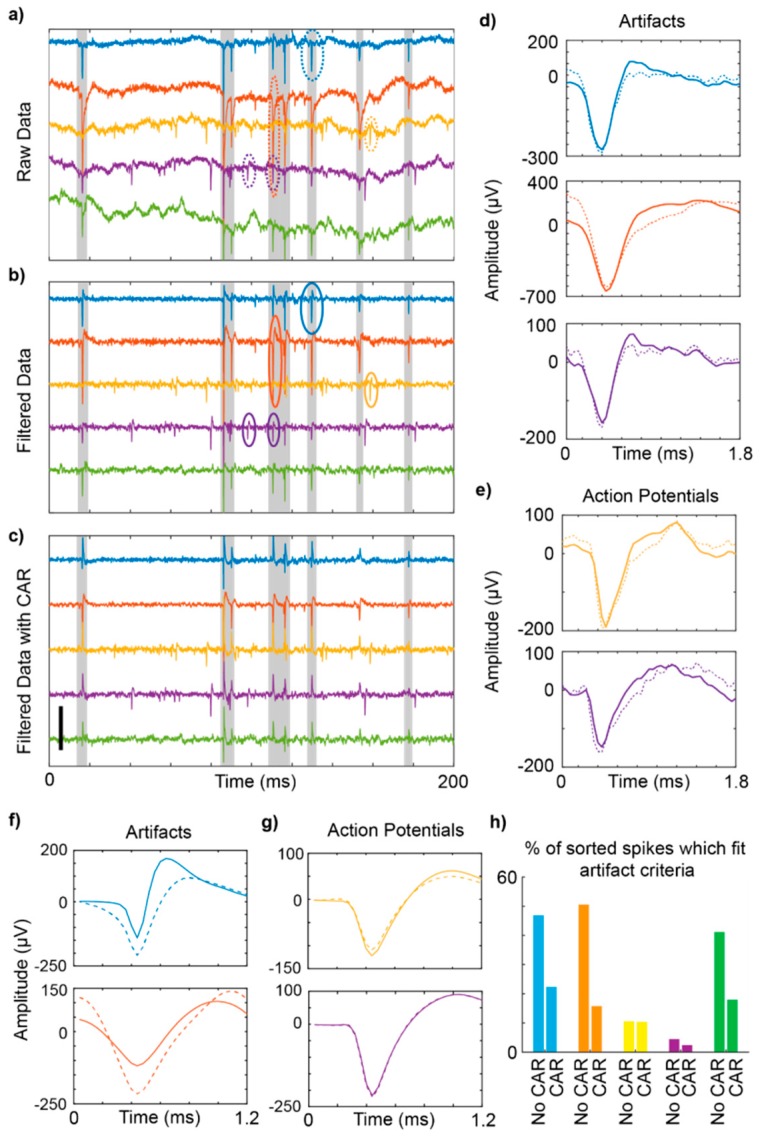
Common average referencing (CAR) helps to reduce motion artifact misclassification. (**a**–**c**) Example data (200 ms) from an awake moving rat, implanted with a linear silicon microelectrode array. Application of a high frequency band pass filter (2nd order Butterworth filter, passband 0.3–1 kHz) can cause artifacts (**a**) to resemble action potentials (**b**). Grey shading highlights artifacts. (**d**,**e**) Circled artifacts and action potentials from (**a**,**b**), shown before and after filtering (dashed and solid lines, respectively). (**f**,**g**) Mean waveforms for clustered artifacts (**d**) and action potentials (**e**) from each channel without CAR (dashed line) and with CAR (solid line). (**h**) Percentage of sorted units which fit the criteria for artifacts defined in the methods. Scalebar: (**a**–**c**) 500 μV. (**a**–**g**): © IOP Publishing. Adapted with permission from [20]. All rights reserved.

Signal processing techniques can alter the temporal characteristics of artifacts and thus further confound the detection and removal of non-neuronal signals. Application of a Butterworth filter to remove local field potentials, for example, may change the shape of movement artifacts via ringing of the filter such that they more closely resemble neuronal action potentials (Figure 6). This issue is particularly noteworthy as some pre-amplifiers are configured with a hardware high pass filter to eliminate slow fluctuations (<2 Hz) that may saturate the amplifier. 

Lastly, changing the electrode material at the electrode/electrolyte interface has been shown to affect the magnitude of the artifact created when the electrode/electrolyte interface is disturbed [17,18,58]. For example, Kahn et al. noted only a 1 mV offset potential when flowing a stream of saline solution across a non-polarizable Ag-AgCl electrode, whereas they observed a 30 mV offset potential when using a polarizable pure silver electrode of similar dimensions [16]. Similarly, it has been demonstrated in vivo in chronic rodent studies that electrode sites coated with poly(3,4-ethylenedioxythiophene) polystyrene sulfonate (PEDOT:PSS) [53] or PEDOT nanotubes [59] exhibit notably less low frequency artifact when compared to control iridium or gold electrode sites in the same animal. 

The data presented here demonstrates that artifact signals may share similar temporal characteristics with relevant neuronal signals and can be erroneously classified as single unit or multiunit events during standard spike sorting operations [20,60] (e.g., thresholding and clustering in principal component space). As the number and timing of neuronal signals has important implications for the transmission of information within the brain, the misclassification of motion artifacts as single or multiunit signals may influence our interpretation of the data, and ultimately our understanding of the underlying neurophysiological processes.

### 4.2. Potential Impact of Differential Recording Strategies

Motion artifact is often minimized in vivo through differential recordings [25]. In a differential recording set-up, the measurements taken from a reference electrode are subtracted from the measurements taken by the working electrode [57]. To eliminate motion artifact, the reference electrode ideally needs to be in a similar location as the working electrode and consist of similar dimensions and material to observe a similar ‘common’ representation of motion artifact for subsequent subtraction [57]. This subtraction is often known as ‘common-mode’ rejection. Electrophysiological recordings in vivo have historically used rigid multi-electrode arrays or tethered microwire systems [9,10,11,25,53,59,61,62], which have several advantages with respect to minimizing motion artifact. First, the rigid structures minimize electrical artifacts by resisting bending, and therefore limit artifacts described above [25]. Second, there are multiple electrodes of similar dimension and electrode material located in the same area of tissue that move in tandem with respect to motion [25]. This maximizes the similarity of recorded motion artifact for subsequent subtraction via common-mode rejection/common average reference strategies [25].

As electrodes on a flexible array can potentially move independently of each other, the representation of artifact on each electrode may differ. For example, electrodes at the most shallow contact and at the deepest contact on a single-shank electrode array would presumably experience different stresses with respect to motion of the brain due to tethering [63]. In turn, the motion artifact on the two contacts would also be different. In this case a common median reference (CMR) [64] may avoid creating spurious artifacts on channels without signal, or a small Laplacian (sLAP) referencing strategy could be employed consisting of electrodes with the most similar representation of motion artifact [20]. Nevertheless, due to the extensive variability in artifact amplitude across channels (Figure 1 and Figure 6), neither common average referencing (CAR), CMR, nor sLAP are likely to be sufficient to completely eliminate the misclassification of artifacts as units.

To minimize spike sorting errors associated with common noise across channels, algorithms that compare signals across channels have been proposed. For example, evaluation of inter-electrode correlation between candidate spike segments as an additional criteria for spike sorting has been shown to improve clustering in principal component space [60,65]. Although this technique reduces the instances of correlated noise impacting the signal, actual units may be discarded with this operation, such as when artifacts occur coincidentally with action potentials or if a neuron’s soma is equidistant from two electrode sites. Additionally, simply removing the corrupted data surrounding artifacts will also eliminate detection of coincident spiking activity. Thus, the problem of successfully identifying and discarding artifactual signals remains an important problem for future investigation. In our data, the principal component of sorted single units maintained their eigenvalues before and after applying a common average reference, while noise typically exhibited dramatic changes in eigenvectors, signal shape, and even signal amplitude (Figure 6f,g). This discrepancy may therefore represent an additional strategy to identify artifacts and is currently under further investigation.

### 4.3. Motion Artifact During Fast Scan Cyclic Voltammetry Measurements Using Carbon Fibers

Like electrophysiological recordings, FSCV neurochemical recordings are also susceptible to motion artifact issues. FSCV recordings are predicated on sweeping a voltage potential through a very small, polarizable carbon fiber electrode in reference to a much larger non-polarizable Ag-AgCl reference electrode to generate a triangle waveform that is repeated at regular intervals [46,47]. The reference electrode must be very large in comparison to guarantee that the impedance of the reference is trivial with respect to the carbon fiber/tissue interface; this in turn guarantees that almost all of the voltage drop occurs at the carbon fiber/tissue interface to drive the oxidation/reduction reaction with electroactive neurochemicals adsorbed to the carbon fiber surface [29,32,40]. The reference is traditionally a non-polarizable electrode to avoid distorting the applied triangle waveform by a change of the potential during the FSCV pulse [20].

During FSCV in vivo, the carbon fiber ‘working electrode’ is implanted in a brain region of interest whereas the Ag-AgCl ‘reference electrode’ is normally placed above cortex. The triangle waveform voltage pulse applied to the carbon fiber electrode with respect to the Ag-AgCl electrode causes electroactive neurochemicals adsorbed to the surface of the carbon fiber to oxidize and reduce, generating a faradaic current. As the carbon fiber is a polarizable electrode, a non-faradaic capacitive current is also generated. The voltage at which oxidation/reduction currents are observed in combination with the magnitude of those currents can be used to infer the specific electroactive neurochemical and the approximate concentration of that neurochemical in the vicinity of the carbon fiber electrode. As the neurochemical concentration undergoes phasic changes, the faradaic current changes while the non-faradaic current remains relatively constant. Therefore, the real-time FSCV waveform can be subtracted by an FSCV measurement at an earlier point in time—known as background subtraction—to minimize the non-faradaic portion of the observed current. This helps isolate faradaic changes in current putatively associated with phasic changes in local neurochemical concentration. 

The idea that spurious signals may contaminate even background subtracted FSCV recordings is not new. The preliminary studies performed by Adams in the 1970s demonstrated that neurotransmitters are only a percentage of species within the brain able to oxidize/reduce when at the electrode surface [30]. This led Mark Wightman and his colleagues to propose a set of guidelines often referred to as the ‘Five Golden Rules’ of electroanalytical chemistry in vivo [28,66]. In brief, these rules include (1) electrochemical verification of the FSCV signal generated by specific concentrations of the neurotransmitter of interest in vitro, (2) anatomical verification that the electrode is placed in a region of the brain in the vicinity of post-synaptic terminals where the neurotransmitter of interest would be found, (3) physiological verification through behavioral manipulation that would be anticipated to change the extracellular concentration of the neurotransmitter of interest, for example electrical stimulation of the pre-synaptic pathway, (4) pharmacological manipulation—e.g., a reuptake inhibitor specific to the neurotransmitter of interest—that would elicit changes in the measured concentrations specific to the neurotransmitter of interest, and (5) independent confirmation with a secondary measure such as microdialysis. Note these rules are predicated on the assumption that measurements of the neurochemical of interest are primarily confounded by interfering signal caused by the adsorption of other electroactive molecules in the brain to the surface of the electrode. In general artifacts due to motion, sources of radio frequency radiation, or electrical/optical stimulation in vivo have previously been thought to create ‘broad-band’ changes in observed currents. These broad-band changes are assumed to be roughly uniformly distributed across all voltages applied during the FSCV pulse [67], instead of changes at the characteristic voltages stereotypical of the oxidation/reduction potentials of a specific electroactive neurochemical. Consequently, datasets contaminated by motion or other artifacts are traditionally identified by a trained operator through visual inspection for broad-band changes and are removed.

Here we have demonstrated that motion can generate an artifact signal during FSCV that resembles phasic neurochemical concentration changes regularly measured in vivo if that neurochemical is present in the recording medium. Motion of the electrode in solution with no electroactive molecules—buffer solution with only salt ions—produces broad band artifact currents that bear no resemblance to specific electroactive molecules. Importantly, changes in current at the oxidation and reduction potential of a neurochemical is defined as a change in concentration of that neurochemical when using FSCV as a measurement tool, therefore measurement of those oxidation/reduction potential current changes during motion is unexpected given the concentration of neurochemical did not change during the motion. 

Motion of the electrode/electrolyte interface during FSCV disturbs the established electric double layer and thus may lead to changes in the capacitive charging current as well as the distribution of charged species, such as dopamine near the FSCV electrode surface [68,69]. In addition, movement of the electrode could change the equilibrium concentrations of dopamine and dopamine-o-quinone (oxidation product of dopamine) near the electrode that is formed as a result of oxidation/reduction, mass transfer, and adsorption dynamics near the electrode [68,70]. Upon motion, the electrode might be leaving the equilibrium formed by those dynamics and entering the bulk concentration that has a higher ratio of dopamine to dopamine-o-quinone, thus resulting in a measured transient increase that decays back to baseline as equilibrium re-establishes. Finally, disturbing either the working or reference electrode transiently alters their respective half-cell potentials. As a voltage is actively being applied between the working and reference electrode to drive redox reactions, disturbing the electrode/electrolyte interface of either may alter the effective voltage being applied between them. 

The finding that motion artifact during FSCV closely resembles phasic changes of electroactive neurochemicals in the solution may be particularly problematic when inferring in vivo concentrations from pre and post-operative calibrations performed in vitro. This issue is compounded by the observation that different neurochemicals produce different motion artifacts, and the magnitude of the motion artifact depends on the tonic concentration of neurochemical in the recording environment. Consequently, commonly used reuptake inhibitors for pharmacological validation—which increase the concentration of the neurochemical of interest in the extracellular environment—may inadvertently also increase the magnitude of motion artifacts that resemble the neurochemical of interest.

It is important to note the motion artifact produced during these experiments does not always exactly resemble the neurochemical’s characteristic redox potentials. For example, a decrease in current was observed at the stereotypical adenosine secondary oxidation peak (1.0 V) during motion (Figure 5, compare to increase at secondary peak for known adenosine in Figure 1). In addition, the magnitude of motion artifact was highly variable given multiple electrodes of the same active site dimensions in a controlled in vitro condition (Figure 4). This suggests motion artifacts may be difficult to predict in magnitude, especially in vivo. It is also important to note that the lowest neurochemical concentrations of dopamine and adenosine used in these experiments are at the high end of what would be anticipated in vivo, with the resulting motion induced current deflections typically in the 1–4 nA ranges. Consequently, the impact of motion artifact on interpreting FSCV becomes more significant in chronic studies where the observed signal is reduced due to biofouling and may be only in the 1 nA range [71,72,73]. 

The findings presented here serve to underscore the necessity of following Wightman’s “Five Golden Rules” when interpreting in vivo FSCV recordings. This includes independent confirmation with a secondary measure, ideally one capable of measuring changes in concentration of the neurochemical of interest, albeit at slower temporal resolution. Other common sources of artifact that disturb the electrode/electrolyte interface, such as electrical stimulation or extrinsic sources of electromagnetic radiation were also preliminarily tested during the execution of these studies (data not shown). Under the right conditions these traditional sources of electrophysiological artifacts also generate characteristic waveforms normally associated with phasic changes of electroactive neurochemicals during FSCV measurements. These perturbations should be explored in future work.

## 5. Conclusions

Both the electrophysiological recording and FSCV motion artifact data presented in this short communication suggest that any change to design or usage-context of a neural interface needs to be carefully evaluated to determine if meaningful neurological signals can be detected and separated from sources of artifact. Validation of meaningful neurological signals ideally includes evaluating a physiological input to drive peri-stimulus neural activity [6,20,31,32,46,47,50,74]. In addition, the stimulus needs to be evaluated on the benchtop or in a post-mortem control to ensure that the stimulus itself does not generate an artifactual signal. Moreover, active shielding and signal processing needs to be carefully evaluated to better identify and remove motion related artifacts as increasingly flexible devices are developed.

## References

[1] Schwartz A.B. (2004). Cortical neural prosthetics. Annu. Rev. Neurosci..

[2] Schwartz A.B., Cui X.T., Weber D.J., Moran D.W. (2006). Brain-controlled interfaces: Movement restoration with neural prosthetics. Neuron.

[3] Kipke D.R., Shain W., Buzsaki G., Fetz E., Henderson J.M., Hetke J.F., Schalk G. (2008). Advanced Neurotechnologies for Chronic Neural Interfaces: New Horizons and Clinical Opportunities. J. Neurosci..

[4] Brandman D.M., Cash S.S., Hochberg L.R. (2017). Review: Human Intracortical recording and neural decoding for brain-computer interfaces. IEEE Trans. Neural Syst. Rehabil. Eng..

[5] Iordanova B., Vazquez A.L., Kozai T.D.Y., Fukuda M., Kim S.G. (2018). Optogenetic investigation of the variable neurovascular coupling along the interhemispheric circuits. J. Cereb. Blood Flow Metab..

[6] Michelson N.J., Kozai T.D.Y. (2018). Isoflurane and Ketamine Differentially Influence Spontaneous and Evoked Laminar Electrophysiology in Mouse V1. J. Neurophysiol..

[7] Golabchi A., Wu B., Li X., Carlisle D.L., Kozai T.D.Y., Friedlander R.M., Cui X.T. (2018). Melatonin improves quality and longevity of chronic neural recording. Biomaterials.

[8] Mosier E.M., Wolfson M., Ross E., Harris J., Weber D., Ludwig K.A. (2018). The Brain Initiative—Implications for a Revolutionary Change in Clinical Medicine via Neuromodulation Technology. Neuromodulation.

[9] Gage G.J., Ludwig K.A., Otto K.J., Ionides E.L., Kipke D.R. (2005). Naive coadaptive cortical control. J. Neural Eng..

[10] Gage G.J., Otto K.J., Ludwig K.A., Kipke D.R. Co-adaptive Kalman filtering in a naive rat cortical control task. Proceedings of the 26th Annual International Conference of the IEEE Engineering in Medicine and Biology Society.

[11] Ludwig K.A., Miriani R.M., Langhals N.B., Marzullo T.C., Kipke D.R. (2011). Use of a Bayesian maximum-likelihood classifier to generate training data for brain-machine interfaces. J. Neural Eng..

[12] Bowsher K., Civillico E., Coburn J., Collinger J., Contreras-Vidal J., Denison T., Donoghue J., French J., Getzoff N., Hochberg L. (2016). Brain–computer interface devices for patients with paralysis and amputation: A meeting report. J. Neural Eng..

[13] Trevathan J.K., Yousefi A., Park H.O., Bartoletta J.J., Ludwig K.A., Lee K.H., Lujan J.L. (2017). Computational modeling of neurotransmitter release evoked by electrical stimulation: nonlinear approaches to predicting stimulation-evoked dopamine release. ACS Chem. Neurosci..

[14] Grahn P.J., Mallory G.W., Khurram O.U., Berry B.M., Hachmann J.T., Bieber A.J., Bennet K.E., Min H.K., Chang S.Y., Lee K.H. (2014). A neurochemical closed-loop controller for deep brain stimulation: Toward individualized smart neuromodulation therapies. Front. Neurosci..

[15] Covey D.P., Garris P.A. Using fast-scan cyclic voltammetry to evaluate striatal dopamine release elicited by subthalamic nucleus stimulation. Proceedings of the 2009 Annual International Conference of the IEEE Engineering in Medicine and Biology Society.

[16] Webster J. (2009). Medical Instrumentation: Application and Design.

[17] Tam H., Webster J.G. (1977). Minimizing electrode motion artifact by skin abrasion. IEEE Trans. Biomed. Eng..

[18] Stecker M. (2017). Factors Affecting Stimulus Artifact: Solution Factors. EC Neurol..

[19] Heffer L.F., Fallon J.B. (2008). A novel stimulus artifact removal technique for high-rate electrical stimulation. J. Neurosci. Methods.

[20] Michelson N.J., Vazquez A.L., Eles J.R., Salatino J.W., Purcell E.K., Williams J.J., Cui X.T., Kozai T.D.Y. (2018). Multi-scale, multi-modal analysis uncovers complex relationship at the brain tissue-implant neural interface: New Emphasis on the Biological Interface. J. Neural Eng..

[21] Wartzek T., Lammersen T., Eilebrecht B., Walter M., Leonhardt S. (2011). Triboelectricity in capacitive biopotential measurements. IEEE Trans. Biomed. Eng..

[22] Giancoli D.C. (1998). Physics: Principles with Applications.

[23] Bard A.J., Faulkner L.R., Leddy J., Zoski C.G. (1980). Electrochemical Methods: Fundamentals and Applications.

[24] Merrill D.R., Bikson M., Jefferys J.G. (2005). Electrical stimulation of excitable tissue: Design of efficacious and safe protocols. J. Neurosci. Methods.

[25] Ludwig K.A., Miriani R.M., Langhals N.B., Joseph M.D., Anderson D.J., Kipke D.R. (2009). Using a common average reference to improve cortical neuron recordings from microelectrode arrays. J. Neurophysiol..

[26] Du Z.J., Kolarcik C.L., Kozai T.D.Y., Luebben S.D., Sapp S.A., Zheng X.S., Nabity J.A., Cui X.T. (2017). Ultrasoft microwire neural electrodes improve chronic tissue integration. Acta Biomater..

[27] Salatino J.W., Ludwig K.A., Kozai T.D.Y., Purcell E.K. (2017). Glial responses to implanted electrodes in the brain. Nat. Biomed. Eng..

[28] Wellman S.M., Eles J.R., Ludwig K.A., Seymour J.P., Michelson N.J., McFadden W.E., Vazquez A.L., Kozai T.D. (2017). A Materials Roadmap to Functional Neural Interface Design. Adv. Funct. Mater..

[29] Patel P.R., Zhang H., Robbins M.T., Nofar J.B., Marshall S.P., Kobylarek M.J., Kozai T.D.Y., Kotov N.A., Chestek C.A. (2016). Chronic In Vivo Stability Assessment of Carbon Fiber Microelectrode Arrays. J. Neural Eng..

[30] Kozai T.D., Jaquins-Gerstl A.S., Vazquez A.L., Michael A.C., Cui X.T. (2015). Brain tissue responses to neural implants impact signal sensitivity and intervention strategies. ACS Chem. Neurosci..

[31] Kolarcik C.L., Luebben S.D., Sapp S.A., Hanner J., Snyder N., Kozai T.D.Y., Chang E., Nabity J.A., Nabity S.T., Lagenaur C.F. (2015). Elastomeric and soft conducting microwires for implantable neural interfaces. Soft Matter.

[32] Kozai T.D.Y., Li X., Bodily L.M., Caparosa E.M., Zenonos G.A., Carlisle D.L., Friedlander R.M., Cui X.T. (2014). Effects of caspase-1 knockout on chronic neural recording quality and longevity: Insight into cellular and molecular mechanisms of the reactive tissue response. Biomaterials.

[33] Kozai T.D.Y., Langhals N.B., Patel P.R., Deng X., Zhang H., Smith K.L., Lahann J., Kotov N.A., Kipke D.R. (2012). Ultrasmall implantable composite microelectrodes with bioactive surfaces for chronic neural interfaces. Nat. Mater..

[34] Guitchounts G., Markowitz J.E., Liberti W.A., Gardner T.J. (2013). A carbon-fiber electrode array for long-term neural recording. J. Neural Eng..

[35] Sohal H.S., Clowry G.J., Jackson A., O’Neill A., Baker S.N. (2016). Mechanical flexibility reduces the foreign body response to long-term implanted microelectrodes in rabbit cortex. PLoS ONE.

[36] Sohal H.S., Jackson A., Jackson R., Clowry G.J., Vassilevski K., O’Neill A., Baker S.N. (2014). The sinusoidal probe: A new approach to improve electrode longevity. Front. Neuroeng..

[37] Harris J.P., Capadona J.R., Miller R.H., Healy B.C., Shanmuganathan K., Rowan S.J., Weder C., Tyler D.J. (2011). Mechanically adaptive intracortical implants improve the proximity of neuronal cell bodies. J. Neural Eng..

[38] Canales A., Jia X., Froriep U.P., Koppes R.A., Tringides C.M., Selvidge J., Lu C., Hou C., Wei L., Fink Y. (2015). Multifunctional fibers for simultaneous optical, electrical and chemical interrogation of neural circuits in vivo. Nat. Biotechnol..

[39] Xie C., Liu J., Fu T.M., Dai X., Zhou W., Lieber C.M. (2015). Three-dimensional macroporous nanoelectronic networks as minimally invasive brain probes. Nat. Mater..

[40] Patel P.R., Na K., Zhang H., Kozai T.D., Kotov N.A., Yoon E., Chestek C.A. (2015). Insertion of linear 8.4 μm diameter 16 channel carbon fiber electrode arrays for single unit recordings. J. Neural Eng..

[41] Ware T., Simon D., Liu C., Musa T., Vasudevan S., Sloan A., Keefer E.W., Rennaker R.L., Voit W. (2014). Thiol-ene/acrylate substrates for softening intracortical electrodes. J. Biomed. Mater. Res. Part B Appl. Biomater..

[42] Nguyen J.K., Park D.J., Skousen J.L., Hess-Dunning A.E., Tyler D.J., Rowan S.J., Weder C., Capadona J.R. (2014). Mechanically-compliant intracortical implants reduce the neuroinflammatory response. J. Neural Eng..

[43] Schluter E.W., Mitz A.R., Cheer J.F., Averbeck B.B. (2014). Real-time dopamine measurement in awake monkeys. PLoS ONE.

[44] Clark J.J., Sandberg S.G., Wanat M.J., Gan J.O., Horne E.A., Hart A.S., Akers C.A., Parker J.G., Willuhn I., Martinez V. (2009). Chronic microsensors for longitudinal, subsecond dopamine detection in behaving animals. Nat. Methods.

[45] Seymour J.P., Kipke D.R. (2007). Neural probe design for reduced tissue encapsulation in CNS. Biomaterials.

[46] Kozai T.D., Catt K., Li X., Gugel Z.V., Olafsson V.T., Vazquez A.L., Cui X.T. (2015). Mechanical failure modes of chronically implanted planar silicon-based neural probes for laminar recording. Biomaterials.

[47] Kozai T.D.Y., Du Z., Gugel Z.V., Smith M.A., Chase S.M., Bodily L.M., Caparosa E.M., Friedlander R.M., Cui X.T. (2015). Comprehensive chronic laminar single-unit, multi-unit, and local field potential recording performance with planar single shank electrode arrays. J. Neurosci. Methods.

[48] Mechler F., Victor J.D. (2012). Dipole characterization of single neurons from their extracellular action potentials. J. Comput. Neurosci..

[49] Henze D.A., Borhegyi Z., Csicsvari J., Mamiya A., Harris K.D., Buzsaki G. (2000). Intracellular features predicted by extracellular recordings in the hippocampus in vivo. J. Neurophysiol..

[50] Kozai T.D.Y., Catt K., Du Z., Na K., Srivannavit O., Haque R.-U.M., Seymour J., Wise K.D., Yoon E., Cui X.T. (2016). Chronic In Vivo Evaluation of PEDOT/CNT for Stable Neural Recordings. IEEE Trans. Biomed. Eng..

[51] De Gennaro L., Ferrara M. (2003). Sleep spindles: An overview. Sleep Med. Rev..

[52] Shahriari K. (2001). Safe and Effective Techniques for Surgically Inserting Flexible Microelectrode Arrays into the Cortex. Ph.D. Thesis.

[53] Ludwig K.A., Uram J.D., Yang J., Martin D.C., Kipke D.R. (2006). Chronic neural recordings using silicon microelectrode arrays electrochemically deposited with a poly(3,4-ethylenedioxythiophene) (PEDOT) film. J. Neural Eng..

[54] Shon Y.M., Chang S.Y., Tye S.J., Kimble C.J., Bennet K.E., Blaha C.D., Lee K.H. (2010). Comonitoring of adenosine and dopamine using the wireless instantaneous neurotransmitter concentration system: Proof of principle. J. Neurosurg..

[55] Swamy B.K., Venton B.J. (2007). Subsecond detection of physiological adenosine concentrations using fast-scan cyclic voltammetry. Anal. Chem..

[56] Jackson J.D. (1999). Classical Electrodynamics.

[57] Webster J.G., Eren H. (2014). Measurement, Instrumentation, and Sensors Handbook.

[58] Kahn A. Motion artifacts and streaming potentials in relation to biological electrodes. Proceedings of the Dig 6th International Conference Medical Electronics and Biological Engineering.

[59] Abidian M.R., Ludwig K.A., Marzullo T.C., Martin D.C., Kipke D.R. (2009). Interfacing conducting polymer nanotubes with the central nervous system: chronic neural recording using poly(3,4-ethylenedioxythiophene) nanotubes. Adv. Mater..

[60] Paralikar K., Rao C., Clement R.S. Automated reduction of non-neuronal signals from intra-cortical microwire array recordings by use of correlation technique. Proceedings of the 2008 30th Annual International Conference of the IEEE Engineering in Medicine and Biology Society.

[61] Ludwig K.A., Langhals N.B., Joseph M.D., Richardson-Burns S.M., Hendricks J.L., Kipke D.R. (2011). Poly(3,4-ethylenedioxythiophene) (PEDOT) polymer coatings facilitate smaller neural recording electrodes. J. Neural Eng..

[62] Purcell E.K., Thompson D.E., Ludwig K.A., Kipke D.R. (2009). Flavopiridol reduces the impedance of neural prostheses in vivo without affecting recording quality. J. Neurosci. Methods.

[63] Subbaroyan J., Martin D.C., Kipke D.R. (2005). A finite-element model of the mechanical effects of implantable microelectrodes in the cerebral cortex. J. Neural Eng..

[64] Rolston J.D., Gross R.E., Potter S.M. Common median referencing for improved action potential detection with multielectrode arrays. Proceedings of the 2009 Annual International Conference of the IEEE Engineering in Medicine and Biology Society.

[65] Paralikar K.J., Rao C.R., Clement R.S. (2009). New approaches to eliminating common-noise artifacts in recordings from intracortical microelectrode arrays: Inter-electrode correlation and virtual referencing. J. Neurosci. Methods.

[66] Phillips P.E., Wightman R.M. (2003). Critical guidelines for validation of the selectivity of in-vivo chemical microsensors. TrAC Trends Anal. Chem..

[67] Garris P.A., Ensman R., Poehlman J., Alexander A., Langley P.E., Sandberg S.G., Greco P.G., Wightman R.M., Rebec G.V. (2004). Wireless transmission of fast-scan cyclic voltammetry at a carbon-fiber microelectrode: Proof of principle. J. Neurosci. Methods.

[68] Johnson J.A., Hobbs C.N., Wightman R.M. (2017). Removal of Differential Capacitive Interferences in Fast-Scan Cyclic Voltammetry. Anal. Chem..

[69] Simakov A.B., Webster J.G. (2010). Motion Artifact from Electrodes and Cables. Iran. J. Electr. Comput. Eng..

[70] Atcherley C.W., Laude N.D., Parent K.L., Heien M.L. (2013). Fast-scan controlled-adsorption voltammetry for the quantification of absolute concentrations and adsorption dynamics. Langmuir.

[71] Nicolai E.N., Trevathan J.K., Ross E.K., Lujan J.L., Blaha C.D., Bennet K.E., Lee K.H., Ludwig K.A. Detection of norepinephrine in whole blood via fast scan cyclic voltammetry. Proceedings of the 2017 IEEE International Symposium on Medical Measurements and Applications (MeMeA).

[72] Schwerdt H.N., Shimazu H., Amemori K.-I., Amemori S., Tierney P.L., Gibson D.J., Hong S., Yoshida T., Langer R., Cima M.J. (2017). Long-term dopamine neurochemical monitoring in primates. Proc. Natl. Acad. Sci. USA.

[73] Singh Y.S., Sawarynski L.E., Dabiri P.D., Choi W.R., Andrews A.M. (2011). Head-to-head comparisons of carbon fiber microelectrode coatings for sensitive and selective neurotransmitter detection by voltammetry. Anal. Chem..

[74] Cody P.A., Eles J.R., Lagenaur C.F., Kozai T.D., Cui X.T. (2018). Unique electrophysiological and impedance signatures between encapsulation types: An analysis of biological Utah array failure and benefit of a biomimetic coating in a rat model. Biomaterials.

